# The Use of Scattering Data in the Study of the Molecular Organisation of Polymers in the Non-Crystalline State

**DOI:** 10.3390/polym12122917

**Published:** 2020-12-05

**Authors:** Thomas Gkourmpis, Geoffrey R. Mitchell

**Affiliations:** 1 Innovation & Technology, Borealis AB, SE-444 86 Stenungsund, Sweden; 2 Centre of Rapid and Sustainable Product Development, Polytechnic of Leiria, 2430-028 Marinha Grande, Portugal; geoffrey.mitchell@ipleiria.pt

**Keywords:** polymer melt, polymer glass, conformational analysis, chain segment packing, neutron scattering

## Abstract

Scattering data for polymers in the non-crystalline state, i.e., the glassy state or the molten state, may appear to contain little information. In this work, we review recent developments in the use of scattering data to evaluate in a quantitative manner the molecular organization of such polymer systems. The focus is on the local structure of chain segments, on the details of the chain conformation and on the imprint the inherent chemical connectivity has on this structure. We show the value of tightly coupling the scattering data to atomistic-level computer models. We show how quantitative information about the details of the chain conformation can be obtained directly using a model built from definitions of relatively few parameters. We show how scattering data may be supplemented with data from specific deuteration sites and used to obtain information hidden in the data. Finally, we show how we can exploit the reverse Monte Carlo approach to use the data to drive the convergence of the scattering calculated from a 3d atomistic-level model with the experimental data. We highlight the importance of the quality of the scattering data and the value in using broad Q scattering data obtained using neutrons. We illustrate these various methods with results drawn from a diverse range of polymers.

## 1. Introduction

Amorphous polymers are characterised by the absence of long-range order, leading to a level of order that is essentially associated with the inherent atomic connectivity and is concentrated in the vicinity of the polymer chain. Consequently, the short-range order of the chain will be dominated by the chemical structure through the spatial organisation of chain segments whose continuous assembly represents the polymer trajectory. The freedom of rotation enjoyed by the chemical bonds leads to a vast array of macromolecular structures, where these continuous assemblies of bonds are able to explore different possible spatial configurations. These chain trajectories are traditionally treated in terms of rotational states that can be discrete or continuous [1]. In principle, large numbers of these states can be considered and the chain segment can be defined as a part of the macromolecule where the chain conformation is assumed to be broadly constant [2].

**Figure 1:**
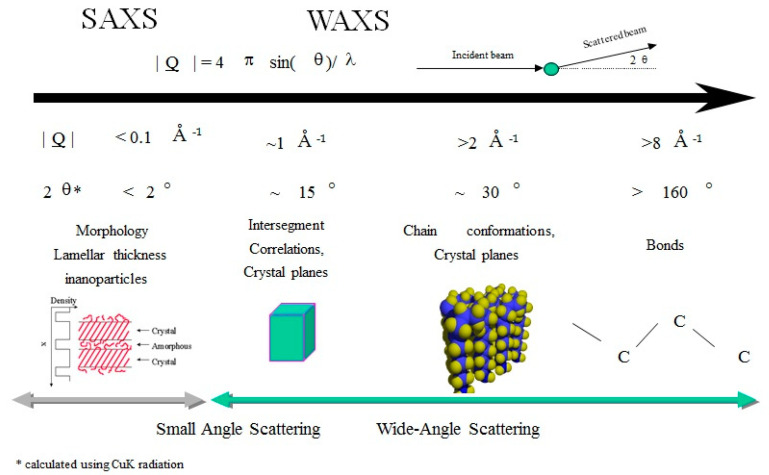
Schematic representation of the different correlations available by the use of diffraction methods in the study of amorphous polymers.

Neutron and X-ray scattering methods are commonly employed in the study of polymeric short-range order due to their sensitivity to the local structural fluctuations. Contrary to NMR methods that provide information only on a very localised level, scattering methods are able to probe a large range of length scales. In the simple case of a disordered system comprised of N identical nuclei, the experimentally measured quantity is the differential cross-section σ, namely the ratio of neutrons scattered in a solid angle element Ω to Ω + dΩ relative to the number of incident neutrons [3,4]. The differential cross-section contains coherent and incoherent components, and most scattering experiments will record the total cross-section. In the case of structural studies as we are discussing here, the component of interest is of coherent nature and can be expressed as

(1) $${\left(\frac{\partial \sigma }{\partial \Omega }\right)}_{coh}=\frac{1}{N}\langle N\overset{N}{\underset{i,j=1}{\sum }}b_{i}b_{j}exp\left(i{\mathrm{Qr}}_{ij}\right)\rangle$$

where *b_i_*, *b_j_* are the coherent scattering lengths of atoms *i* and *j*, respectively, *N* is the total number of atoms and r*_ij_* is the position vector between atoms *i* and *j*. The angular brackets indicate the thermal average and Q is the scattering vector commonly expressed as Q=4πsinθ/λ, where 2θ is the scattering angle and λ is the incident wavelength [3,4]. In a neutron scattering experiment over a broad *Q* range, we can obtain information about bond lengths and segmental interactions at distances of approximately 0.1–100 Å. The experimentally observed structure factor *S*(Q) is defined as the spatial Fourier transform of the atomic pairwise correlation functions

(2) $$S\left(Q\right)=1+\rho_{o}\int_{\infty }^{0}\left(g\left(r\right)-1\right)exp\left(i\mathrm{Qr}\right)dr$$

with *ρ_o_* the average density of the system and *g(r)* the pair distribution function that quantifies the probability density of an atom existing at a distance **r** from the origin. In the case of multiple chemical species, both *S*(Q) and *g(r)* can be split into partial terms. The experimentally observed structure factor arises from a wide range of structural correlations in the bulk. From this expression, we can see that structural correlations can be separated into those arising from correlations within the chain (intrachain) and those arising by different chains (interchain) [2,5,6]. The interchain correlations provide information on the different ways polymeric segments arrange themselves in the bulk. The intrachain contribution to the scattering can be extracted from the observed structure factor at values of the momentum transfer Q≥2−2.5 Å^−1^, using atomistic modelling techniques [6]. In Figure 1, a schematic representation of the different structural regimes accessible for neutron and X-ray scattering can be seen.

Traditionally, most experimental studies on glassy and liquid polymers focus on large-scale properties probed by small-angle scattering [7] and dynamics of the glass transition [8]. Studies on amorphous polymers probing the local conformation over extended *Q* ranges have been made mostly by X-rays [9], and the advantage of neutrons to extend the available reciprocal space has not been used to the same extent. Conventionally, computer simulations are used to extract information regarding the different correlations and conformations via comparison with diffraction data. Due to the complex nature of the systems under investigation, a computational model tends to simplify the details of the chemical characteristics, in favour of the long-range interactions. In techniques such as molecular mechanics and molecular dynamics, the overall target is a realistic representation of the thermodynamic and macroscopic properties of the material under investigation [10]. Therefore, the chain conformation is generally represented in terms of multiple-body intramolecular potentials that relate to bond stretching, valence angle bending, and dihedral rotations. Nonbonded interactions are treated via van der Waals and electrostatic interactions. The main shortcoming of these techniques is that despite the fact that the force field is obtained via highly accurate ab initio calculations or experiments on small molecules, it does not always provide the necessary detailed description of a complex polymeric system [11].

The reverse Monte Carlo (RMC) method is a deceptively simple approach that allows for the extraction of detailed atomistic structural parameters of liquids and solids from diffraction data [12,13]. A number of variations of the base method has been proposed since its introduction, dealing with specific types of disordered materials, and more has recently been extended to incorporate the existence of crystals within the structure [13,14]. In this work, we will discuss the advantages of neutron scattering at small angles and deuterium labelling, and how this offers an approach to unlocking the secrets of the chain conformation and revealing the polymer structure on a multitude of length scales. At the same time, we will discuss the role of the intimate coupling of the experimental data with computer-generated models that are centred on a range of different approaches originating from the RMC method that allows for the creation of realistic three-dimensional models of amorphous and semi-crystalline polymers.

## 2. Diffraction and Experimental Requirements

Most neutron diffraction experiments over extended *Q* ranges are total scattering experiments, meaning that all neutrons that reach the different detector banks are considered. The existence of hydrogen due to its incoherent cross-section (or scattering length) can cause significant problems due to the substantial ‘background’ signal which contains no structural information. One of the most direct solutions to this problem is the substitution of hydrogen atoms with deuterium that allows contrast variation and minimises the incoherent scattering from the sample [3,4,15].

Diffractometers with monochromatic incident beams are able to extend the *Q* range by use of short wavelengths and high scattering angles. In a pulsed neutron experiment, a relatively large number of epithermal neutrons of short wavelengths are available, something that is usually not possible for traditional reactor setups. These short-wavelength neutrons allow a wide range of *Q* values to be explored at relatively small angles, leading to more straightforward corrections of inelasticity effects that are further reduced with scattering angle [16]. A prime example of instruments that take advantage of these possibilities by scanning all scattering angles with a continuous range of detectors is SANDALS [17,18] and NIMROD [19,20] diffractometers at the ISIS Pulsed Neutron Source. The main disadvantage of using detectors at small angles is the reduced resolution ΔQ/Q, but for most instruments, a resolution of the order of ~2.5–4% is adequate for the majority of studies of disordered materials. Here, we must note that X-ray diffractometers tend to be limited due to the incident wavelength used. Moreover, the extent of Compton scattering increases greatly at higher *Q* and so tends to generate problems unless removed experimentally (e.g., as was the case of the 9.1 station at the Daresbury SRS) [21,22] or using an energy-dispersive detector [23]. The latter is limited in usage to MoKα in a laboratory environment. For these reasons, we chose to focus on neutron scattering, although the same principles and methodology could be applicable to X-rays.

## 3. Use of Partial Structure Factors

In the case of multicomponent disordered materials, the experimentally observed structure factor *D*(Q) can be expressed as

(3) $$D\left(Q\right)=\underset{\alpha ,\beta \geq \alpha }{\sum }\left(2-\delta_{a\beta }\right)c_{a}c_{\beta }b_{a}b_{\beta }S_{a\beta }\left(Q\right)$$

with *S_αβ_* the partial structure factors with weights determined by the products of the atomic fractions *c_α_*, *c_β_* and the scattering length. The Kronecker delta *δ_αβ_* is introduced to avoid multiple counting of similar items. According to this, the partial structure factor can be related to the appropriate partial distribution function *g_αβ_(**r**)* by

(4) $$S_{\alpha \beta }\left(Q\right)=\rho \overset{\infty }{\underset{0}{\int }}g_{\alpha \beta }\left(r-1\right)exp\left(i\mathrm{Qr}\right)dr$$

If the set of materials used is isostructural, then a set of total diffraction patterns *D*(Q) can be used to produce a complete set of partial structure factors accompanied by the associated radial distribution functions [5].

**Figure 2:**
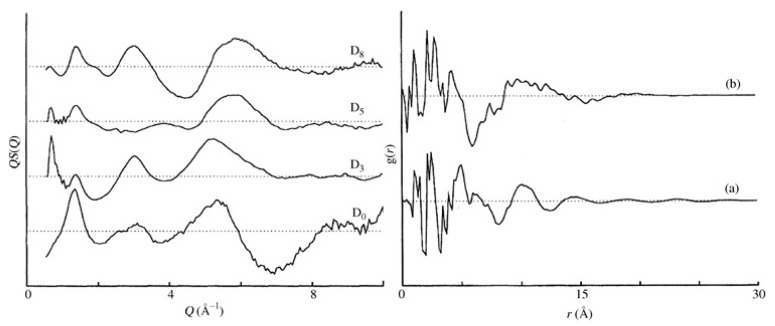
Structure factors (left) and radial distribution functions (right) for selectively deuterated glassy polystyrene. The level of deuteration can be assessed by the number next to the label B (see text for full details). (a) indicates the total correlation function for system D_8_ and (b) the partial correlation function *g_bb_* describing the correlations involving only the backbone atoms. Both graphs were reproduced from reference [2].

These partial structure factors can be obtained from computational models [24,25] or directly from the experiments themselves [5]. For a system of atactic polystyrene (a-PS), the scattering can be distinguished between that which originates from the side groups and that originating from the backbone atoms. Consequently, these structural correlations can be represented via partial correlation functions related to interactions between specific sites $g_{bb}\left(r\right)$, $g_{bs}\left(r\right)$ and $g_{ss}\left(r\right)$ with *b* and *s* indicating the backbone and side group, respectively. These partial correlation functions can be probed via selective substitution of hydrogen with deuterium, and high-quality neutron scattering data were collected for four glassy polystyrene samples D_0_, D_3_, D_5_ and D_8_, with the number indicating the level of deuteration with respect to the monomer unit [2]. D_3_ involves the deuteration of the skeletal chain, whereas D_5_ refers to the deuteration of the side group. The existence of different deuteration levels leads to significant changes in the observed structure factor, something that is not visible with X-rays. Further manipulation of these functions leads to a set of partial correlation functions (see Figure 2).

These partial correlation functions indicate the existence of a broad feature at r ~ 10–10 Å that is not visible in the total correlation function obtained from D_8_. This effect has been attributed to the correlation between polymer backbones, and the peak is fairly broad and weak, indicating very limited backbone correlations. Such a feature is much weaker than the corresponding features in simple polymers like polyethylene [26] or poly(tetrafluoroethylene) [27], thus concluding that no substantial chain correlations are present in glassy polystyrene [2]. These limited backbone correlations further indicate that correlations between the bulky side groups dominate the structure, a model previously proposed on the basis of X-ray scattering analysis [28].

## 4. Local Mixing in Polymer Blends

Simple theory assumes that mixing between polymers is random at the segmental level, although experimental evidence suggests otherwise. It has been shown that the miscibility of polyolefins is essentially controlled by the values of their solubility parameters that can be determined by small-angle neutron scattering (SANS) measurements [29]. Solubility parameters can also be derived directly from knowledge of chain dimensions usually expressed via the packing length. In other words, the local chemical topology (branches, unsaturated bonds, specific interactions, etc.) is expected to play a very strong role in the local mixing process, something seen in the case of mixtures of chemically similar but topologically dissimilar polymers like 1,4 and 1,2 polybutadiene [30].

Let us consider blends of deuterated and protonated 1,2 and 1,4 polybutadiene, namely d-14pbd/d-12pbd, d-14pbd/h-12pbd, h-14pbd/d-12pbd and h-14pbd/h-12pbd where the d or h indicate a deuterated or protonated system, respectively. For a binary mixture, we can express the interchain component in terms of partial structure factors $P_{AA}\left(Q\right)$, $P_{AB}\left(Q\right)$ and $P_{BB}\left(Q\right)$ where A and B are segments of the two chemically different polymers. According to this, the total structure factor can be expressed as

(5) $$S_{i}\left(Q\right)=c_{A}c_{A}F_{A_{H,D}}^{2}P_{AA}\left(Q\right)+2c_{A}c_{B}F_{A_{H,D}}F_{B_{H,D}}P_{AB}\left(Q\right)+c_{B}c_{B}F_{B_{H,D}}^{2}P_{BB}\left(Q\right)$$

with *c_A_*, *c_B_* the concentrations of segments *A* and *B*, respectively, and $F_{A}\left(Q\right)$, $F_{B}\left(Q\right)$ the molecular form factors for the given segments. Here, we must note that these expressions are valid for samples that are isotropic in which the local mixing is also isotropic. Extracting the partial structure factors from such an expression requires a series of isostructural blends where either composition of the deuteration level (that alters the molecular form factors) is systematically varied. This approach can only be carried out with neutron scattering methods, as X-ray diffraction is insensitive to the isotope content, thus confirming that the blends are largely at least isostructural.

**Figure 3:**
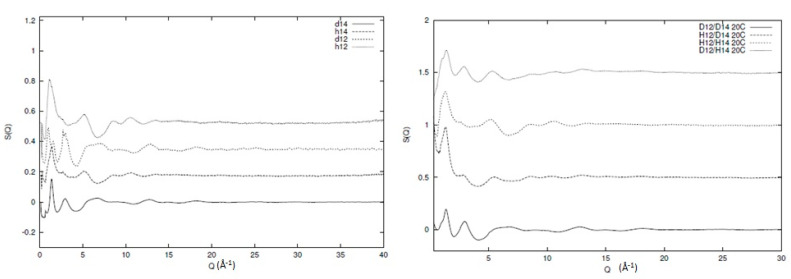
Experimentally observed structure factors of protonated and deuterated 1,2 and 1,4 polybutadiene (left) and blends consisting of these components (right). In both graphs, the term d or h indicate the deuterated or protonated system, respectively, and the term 1,2 or 1,4 indicate the stereoregularity of the polybutadiene chain, respectively.

**Figure 4:**
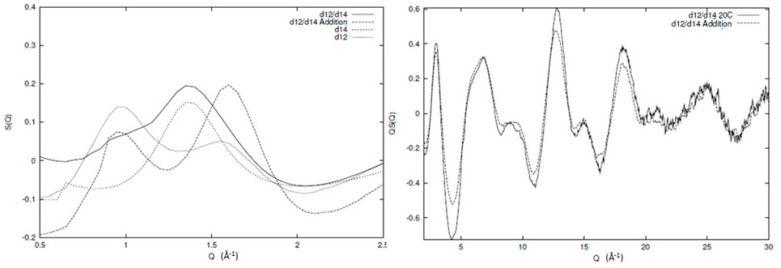
Comparison of the experimentally observed structure factor for a blend of deuterated 1,2 (d12) and 1,4 (d14) polybutadiene (d12/d14) with an artificial structure factor (d12/d14 addition) comprising the sum of the experimentally observed structure factors of the two individual components weighted by their relevant concentration. See text for more detailed discussion.

In Figure 3, the broad Q neutron scattering results for two of the individual components (protonated and deuterated 1,4 and 1,2 polybutadiene) and the resulting blends can be seen. Information regarding the sample preparation and experimental procedure for the individual components can be found elsewhere [6,31] and for the blends in the Supportive Information. Clearly, qualitative differences between the different structure factors exist (as expected) but the data quality and extent of Q range are visible. In Figure 4, we can see the inter and intra chain parts of one of the blends (d12/d14) as an illustration of the overall behaviour of all the different components. The main peak in the structure factor arises from correlations between chain segments, and is sensitive to the level of mixing. Therefore, if the system is to be totally immiscible, then the main peak will be the addition of the two peaks of the individual components weighted by their appropriate contributions. From Figure 4, we can see that this is clearly not the case, as the artificial structure factor (d12/d14 addition) that has been created by simple addition of the individual components is not the same as the observed structure factor for the blend. In other words, a certain level of mixing is present in the system and the decomposition with respect to the partial structure factors is, to a large extent, valid. Another important element we see from Figure 4 is the intrachain scattering that is very similar to the simple addition of the intrachain scattering of the individual components, indicating that the local conformation remains largely unchanged during mixing.

**Figure 5:**
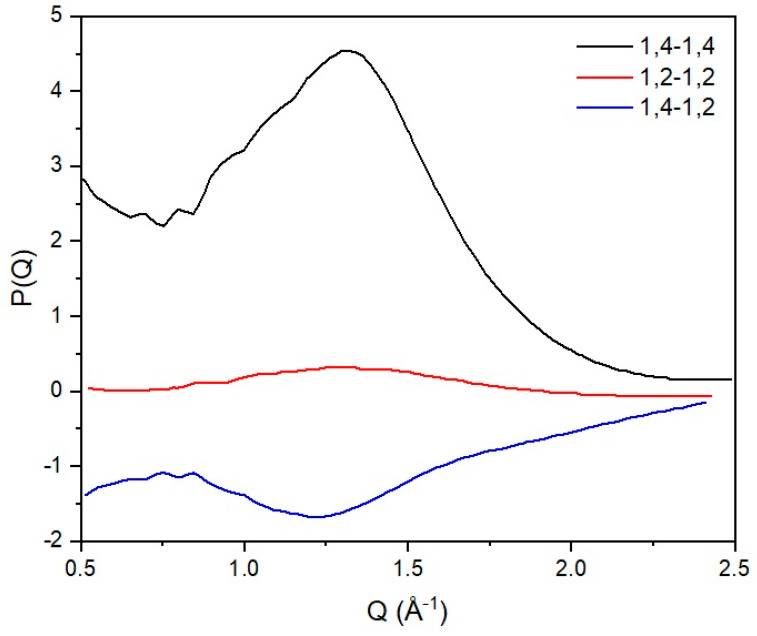
Partial structure factors for the different correlations derived from experimental data on blends of 1,2 and 1,4 polybutadiene. 1,2-1,2 (red), 1,2-1,4 (blue) and 1,4-1,4 (black) indicate the correlations between segments of 1,2 and 1,4 polybutadiene respectively. See text for more detailed discussion.

In this work, the interchain peak is of interest and, in our approach, we decompose the observed structure factor into inter and intrachain components. We have developed atomistic models that match the intrachain scattering for 1,4 [6] and 1,2 polybutadiene [31], and this is subtracted from the observed structure factor yielding the scattering that arises from the intersegmental scattering alone. The partial structure factors can then be obtained by inversion of the previous expression, and the results can be seen in Figure 5. From this analysis, we can clearly see that there is very limited mixing at the local level, as indicated by the cross-term (1,2–1,4). From the width of the interchain peak, we can estimate that the extent of spatial correlation between chemically similar segments is limited to ~2 chain segments.

Broad Q neutron scattering on isotopically labelled blends provides a route to extracting partial structure factors that arise from correlations between chain segments belonging to the same or different polymers, thus allowing a level of quantification in the extent of local mixing in a blend. This is critical as it allows the quantification, mapping, and insight into a number of different factors that are condensed into a single interaction parameter in the conventional Flory–Huggins theory and its variations [32]. In the case of polybutadiene-based blends considered in this work, it has been found that the very local state is largely unmixed in contrast with larger length scales at which the system appears homogeneous and optically clear.

## 5. Coupling Diffraction with Modelling

**Figure 6:**
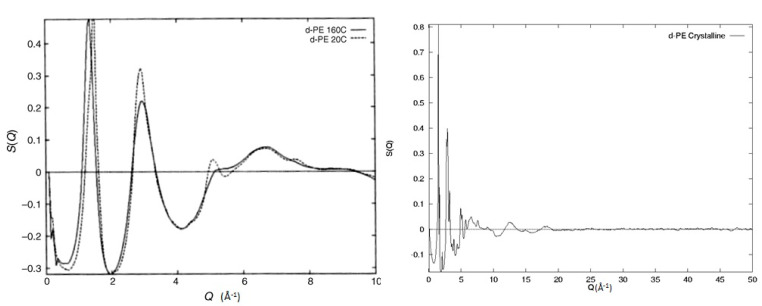
Experimentally observed structure factors from samples of deuterated polyethylene prepared with different methods. The solid line indicates the sample irradiated in the melt (160 °C), and the dashed line indicates the sample irradiated at room temperature (20 °C). On the right-hand side, the diffraction of the semi-crystalline deuterated polyethylene can be seen.

From the discussion so far, it is clear that the structure factor contains a considerable amount of structural information, making the use of molecular modelling an attractive option for probing all these length scales. Before jumping to the modelling techniques though, some important considerations need to be made. By comparing the experimental data to the model, how do we quantify the possible differences? What do these say about the model used? Does this mean that the model is totally wrong or other ways of introducing and/or analysing segmental orientations are available? Without information of this sort, small deviations between observed and predicted structure factors may be vitally important or just trivial computational limitations (e.g., size of the model). Without a concrete and robust understanding of the level of information available from the experiment and how this information is coupled with the model, any further validation is based on uncertain foundations. In Figure 6, we can see an example of such a case where two structure factors of amorphous deuterated polyethylene (d-PE) prepared with different methods are shown. The experimental procedure was similar to the one used for the polybutadiene samples discussed earlier and can be found in the Supportive Information. The first sample is molten polyethylene irradiated at 160 °C with high-energy electrons (1 MeV, 50 MGy) that induce both cross-linking and chain scission [33]. The observed structure factor is similar to the one observed for molten polyethylene [26]. The second sample has been prepared by extensive irradiation in the absence of oxygen by high-energy electrons (1 MeV, 50 MGy) of semi-crystalline polyethylene at 20 °C. The irradiation of crystalline polyethylene with doses greater than 30 MGy is known to lead to the destruction of crystals [34]. The two structure factors seen in Figure 6 exhibit no Bragg peaks and are similar to the ones expected from amorphous materials. Still, they do exhibit some clear differences, indicating that these two amorphous structures are different. The feature at ~5 Å^−1^ for the 20 °C sample is indicative of extensive trans sequences along the backbone [35] with the interchain peak maximum occurring at Q = 1.5 Å^−1^ in comparison with Q = 1.35 Å^−1^ for the sample irradiated in the melt, suggesting a closer packing of chains. From this superficial analysis of the structure factor, we can deduce that the sample irradiated at 20 °C probably contains some small clusters of parallel segments in the trans configuration while the sample irradiated in the melt is representative of a more random coil. In other words, these two structure factors represent two totally different models of orientation correlations despite the experimentally observed broad similarities. This example is quite informative and underlines the need for detailed, robust, and accurate models and the level of sensitivity of the different structural parameters on the overall structure factor.

## 6. Real vs. Reciprocal Space Functions—The Reverse Monte Carlo Method

The patterns shown in Figure 2 contain the same information, but it is obvious that the level of information that is pronounced is different between the data representation in real (Figure 2 right) and reciprocal space (Figure 2 left). The real-space representation relates with the high level of accuracy to distances that are determined via covalent bonding. Larger distances associated with spatial arrangements and rotations such as valence and torsional angles are far more difficult to extract, making the identification of the local conformational characteristics a very challenging affair. An alternative to such problems is the use of the reciprocal space function as obtained by the experiment. This can be done by ‘reconstructing’ the radial distribution function using a computer model and comparing the calculated with the observed scattering pattern S*_E_*(Q). The calculation of the scattering from a model S*_C_*(Q) can be obtained using Debye’s relationship [6].

(6) $$S_{C}\left(Q\right)=\frac{1}{N}\underset{i}{\sum }\underset{i\neq j}{\sum }b_{i}b_{j}\frac{\sin\left({\mathrm{Qr}}_{ij}\right)}{{\mathrm{Qr}}_{ij}}$$

where *N* is the number of atoms, *b_i_*, *b_j_* are the neutron scattering lengths [3,4] of atoms *i* and *j*, respectively, and r*_ij_* is the equivalent interatomic distance. We have chosen to make the comparison using the reciprocal- instead of the real-space function due to the simplicity of separating inter and intrachain contributions.

The computer-generated structure factor is easily obtained as all the atomic coordinates are known; therefore, by such reconstruction of the scattering and subsequent comparison with the actual experimental data, it allows a vast volume of structural parameters to be probed and their influence in the overall scattering to be established.

### The RMC Method

The RMC technique is based on the traditional Metropolis Monte Carlo (MC) method [36] and aims to generate an atomistic configuration (model) that is consistent with an experimentally observed data set within its errors and is subject to a set of pre-defined constraints. All errors are assumed to be statistical and have a normal distribution [13].

In the general case, we assume N nuclei placed randomly in a cell under periodic boundary conditions. As all the positions of the atoms are known, we can calculate the partial radial distribution function from the given configuration

(7) $$g_{\alpha \beta }^{C_{o}}\left(r\right)=\frac{n_{\alpha \beta }^{C_{o}}\left(r\right)}{4\pi r^{2}dr\rho c_{\alpha }}$$

where *ρ* is the atomic density, *c_α_* is the concentration of nuclei of atom *α* and $n_{\alpha \beta }^{C_{o}}\left(r\right)$ is the number of nuclei of type *β* located at a distance between r and r + dr from the nuclei of type *α*, averaged over all nuclei of type *α*. By performing a Fourier transform on $n_{\alpha \beta }^{C_{o}}\left(r\right)$, we can obtain the partial structure factor $F_{\alpha \beta }^{C_{o}}\left(Q\right)$ and the total structure factor $S^{C_{o}}\left(Q\right)$:

(8) $$F_{\alpha \beta }^{C_{o}}\left(Q\right)=\rho \overset{\infty }{\underset{0}{\int }}4\pi r^{2}\left(g_{\alpha \beta }^{C_{o}}\left(r\right)-1\right)\frac{\sin Qr}{Qr}dr$$

(9) $$S^{C_{o}}\left(Q\right)=\sum^{}c_{\alpha }c_{\beta }b_{\alpha }b_{\beta }\left(F_{\alpha \beta }^{C_{o}}\left(Q\right)-1\right)$$

where *Q* is the momentum transfer and $b_{\alpha }b_{\beta }$ are the coherent neutron scattering lengths for nuclei *α* and *β*, respectively.

At this moment, we have two structure factors, that obtained by the experiment $S^{E}\left(Q\right)$ and that obtained by the atomistic model $S^{C_{o}}\left(Q\right)$. In order to test the accuracy of our model, we need an impartial way of comparing the two structure factors. This can be done via a statistical *χ*^2^ test:

(10) $$\chi_{o}^{2}=\overset{m}{\underset{i=1}{\sum }}\left(\frac{{\left(S^{C_{o}}\left(Q_{i}\right)-S^{E}\left(Q_{i}\right)\right)}^{2}}{\sigma^{2}\left(Q_{i}\right)}\right)$$

With the sum taken over *m* experimental points, σ corresponds to the experimental error. Furthermore, we note that the minimum *Q_i_* value needs to be larger than 2π/L with *L* corresponding to the minimum dimension of the configuration under investigation.

As soon as the initial (old) configuration is compared with the experimental one, we can move one atom at random (limitations on the maximum distance that these move are allowed to exist, but this is beyond the scope of this work [12,13]). Due to this move, a new configuration is available and, thus, a new pair distribution function $g_{\alpha \beta }^{C_{n}}\left(r\right)$, followed by a new partial $F_{\alpha \beta }^{C_{n}}\left(Q\right)$ and total $S^{C_{n}}\left(Q\right)$ structure factor, respectively. As with the old configuration, we can again calculate the accuracy of the new configuration against our model via the new *χ*^2^ test:

(11) $$\chi_{n}^{2}=\overset{m}{\underset{i=1}{\sum }}\left(\frac{{\left(S^{C_{n}}\left(Q_{i}\right)-S^{E}\left(Q_{i}\right)\right)}^{2}}{\sigma^{2}\left(Q_{i}\right)}\right)$$

If $\chi_{n}^{2}<\chi_{o}^{2}$, the move is accepted and the new configuration becomes the old one. If $\chi_{n}^{2}>\chi_{o}^{2}$, the move is accepted with a probability $exp\left(-\left(\chi_{n}^{2}-\chi_{o}^{2}\right)/2\right)$; otherwise, it is rejected. The procedure continues until no further changes below a pre-defined limit can be observed in the value of the statistical test. In practice, as the calculated configuration approaches the experimentally observed one, the value of the statistical test reaches an equilibrium and oscillates around a mean value. The configuration that corresponds to the minimal statistical test is a three-dimensional structure that is consistent with the experimental data within the relevant experimental error. In practice, both the radial distribution function and the structure factor can be used for fitting, although the use of the reciprocal space functions has been seen to be more sensitive to small alterations of the chain conformation [6].

In an RMC process, the quantity that is sampled is equivalent to the sampling quantity of the Metropolis Monte Carlo $\left(U_{n}^{2}-U_{o}^{2}\right)/kT$ with U the potential energy of the configuration for a given interatomic potential, T the temperature and k the Boltzmann constant. In other words, the structure factor can be considered the driving force of the model (i.e., energy), and σ corresponds to the temperature. In principle, any type of statistical test for comparison of that observed with the simulated structure factor can be used, but the χ^2^ is typically used due to simplicity [6,13,31]. It can be shown that for a structural system with only pairwise forces, the RMC procedure leads to an exact solution.

#### RMC Variations

The RMC protocol discussed previously has been used with various degrees of success in the study of the atomic structure of disordered materials like simple liquids [37], molten salts [38], glasses [39] and polymers [26,27]. The basic idea of the method, namely the comparison of a computer-generated model with experimental data following a stochastic set of pre-defined rules, has been extended to approaches that treat the intrachain contribution [6,31], the establishment of actual experimentally based force fields [40] and the role of crystallinity in the overall structure [14,41]. Extending the simulation towards coarser length scales and introducing crystals by arranging the segments in specified ways while keeping them consistent with the chain conformation have been seen to offer possibilities for an in-situ study of time-resolved crystallization [42,43], thus indicating the potential of such intimate coupling of an RMC-based procedure with experimental data [44,45].

## 7. The Role of the Scattering Data

Based on our previous discussion, we can clearly see that the extent, resolution and quality of the experimentally observed structure factor will have an influence on the outcome of the RMC modelling procedure. In this section, we will discuss the role of the diffraction pattern on the computational model and provide some examples on a number of different polymeric systems.

### 7.1. Intrachain Correlations

In this section, we will discuss a variation in the traditional RMC procedure that focuses on the extraction of structural information regarding the intrachain correlations and the chain conformation. In this procedure, we utilise only part of the experimental data that corresponds to the intrachain contribution to the diffraction pattern. The model can be generated by using internal coordinates for the definition of the polymer chain in terms of bond lengths, valence, and torsion angles with values assigned in each parameter through a stochastic Monte Carlo procedure where the relevant parameters are drawn from probability distributions representing the possible value ranges [6]. In most cases, the distributions used are Gaussian, but this is not exclusive and, if necessary, other more complex distributions can be used. Using this simple procedure, we can scan through a large number of parameters and, in order to acquire good statistics, we can average over a number of different models (usually 100). Each parameter representing a probability distribution is varied in a systematic way using a grid method to find the values that minimise the statistical test.

**Figure 7:**
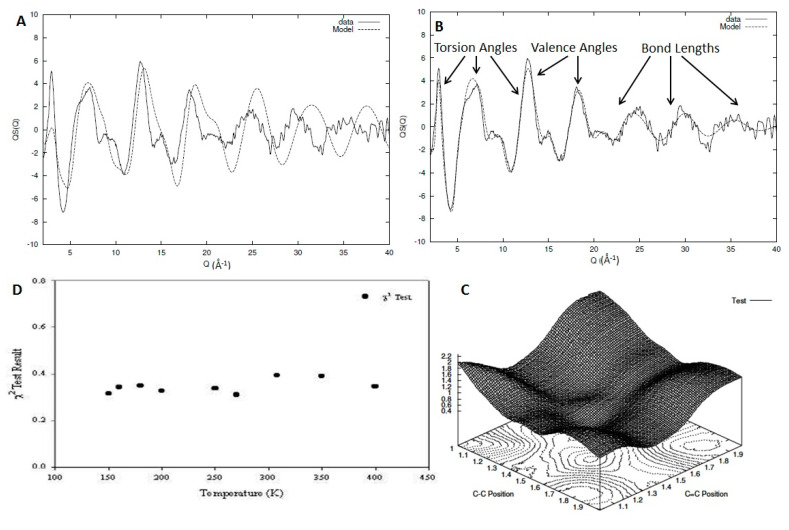
Comparison between the experimentally observed structure factor (solid line) and that calculated from a model (dashed line) for 1,2 polybutadiene at 250 K (**A**,**B**). In (**B**), the areas where the different structural parameters have the most impact on the structure factor is indicated. In (**C**), a surface plot of the statistical test as a function of the C-C and C=C bond lengths can be seen for a model of 1,4 polybutadiene. In (**D**), the final result of the best-fit statistical test for the different models of 1,2 polybutadiene can be seen.

In Figure 7A,B, we can see the intrachain part of the experimentally observed structure factor for a system of deuterated 1,2 polybutadiene as compared with the initial (A) and final (B) model after the refinement has been completed [31]. Using these types of iterative methods allows us to identify the impact different types of structural parameters and their resulting correlations have on the structure factor (Figure 7B). In Figure 7C, we can see an example of the parameter search through the multidimensional space with a plot of the statistical test against the values of the C-C and C=C positions (lengths) for the case of 1,4 polybutadiene [6]. This method has been tested for a series of high-quality data for 1,2 and 1,4 polybutadiene for a wide range of temperatures covering almost all the experimentally accessible range, allowing us to obtain information both in the liquid and glass state [6,31]. In other words, each temperature of each material (9 for 1,2 polybutadiene and 11 for 1,4 polybutadiene) can be considered an independent measurement that can be used to validate the model. In Figure 7D, we can see the statistical test result for the optimal model structure for each of the temperatures for the sample of 1,2 polybutadiene. Although some variation exists, we can clearly see that all the *χ*^2^ results are clustered around the same mean value, indicating the robustness and limitations of the method.

### 7.2. The Case of Selenium

**Figure 8:**
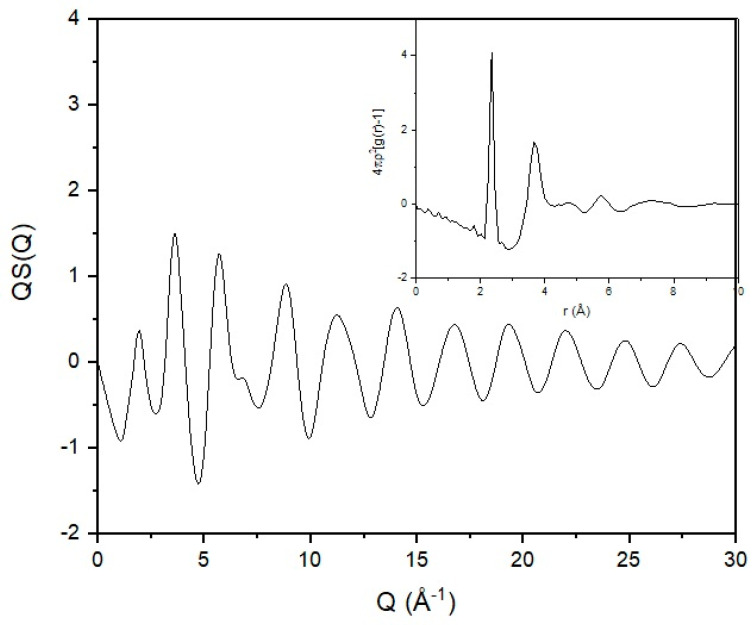
Total structure factor of vitreous selenium obtained from neutron scattering data. In the inset, the radial distribution function of the same scattering curve can be seen.

In order to highlight the role of the experimental data and their influence on the modelling output, we will use a set of neutron scattering data for vitreous selenium. Selenium was considered by Flory as an example of a simple chain in having a one-bond repeat [1]. In Figure 8, the total structure factor for a sample of vitreous selenium can be seen. Sample preparation and experimental details can be found elsewhere [46,47]. A series of broad and diffuse peaks indicating the absence of any crystalline features are present in the scattering pattern. Observation of experimental structure factors [48,49] and computational predictions [50] show that the position and width of the first intense peak changes with temperature. The rest of the scattering curve remains though practically unaffected. This difference can be attributed to the different nature of correlations probed by the different parts of the scattering curve. The first intense peak arises from the packing of selenium in the bulk, and it is of intermolecular origin. The rest of the curve is thus probing interactions of intramolecular origin.

As discussed previously, structural information carried by the diffraction pattern can be extracted via the use of real-space functions through the reduced correlation function and the radial distribution function [6]. Structural investigation in real space has the advantage of locating with high accuracy near-neighbour distances as they appear in the form of sharp and well-defined peaks. Consequently, the level of information probed from a broad Q scattering experiment is on the atomic connectivity and co-ordination. This can be achieved by deconvolution of the real-space function to its constituent components [46]. From the radial distribution function (inset in Figure 8), we can see a very well-defined first sharp peak that indicates the near-neighbour distance. Correlations seem to be weak at distances above 10 Å in agreement with previously reported experimental results [48] and computational predictions [51].

The mean value for the near-neighbour distance has been found to be 2.346 ± 0.013 Å, in good agreement with previously reported values [52,53,54,55]. The coordination number was found [47] to be 1.974 ± 0.205, in good agreement with previously reported values [55,56,57]. The mean value of 1.974 for the coordination number is very close to the ideal value of 2. Having a coordination number of 2 (or close to 2) fits both chains and rings in the amorphous selenium structure. As the value extracted is less than 2, it indicates that undercoordinated atoms (chain ends) exist in the structure. The second peak has a maximum at ~3.75 Å [47], in good agreement with previously reported experimental values [52,53,54,55]. This peak is believed to arise from the superposition of the second near-neighbour interactions and intermolecular correlations [54]. The coordination number was found [47] to be ~6.7, indicating the existence of two second neighbours (covalently bonded to the first neighbours) and elements of intermolecular (in terms of rings or chains) interactions caused by van der Waals forces. The valence angle was calculated to be located at 104° with a RMS deviation of 5° in good agreement with previously reported values [58].

Due to the weak diffuse peaks following the near-neighbour one, it is very difficult to extract information concerning the torsional rotation from the radial distribution function. The superposition of the sine waves of the Fourier transform leads to a series of broad and diffuse peaks that, although they carry information concerning the valence angles and the torsion rotation, it is challenging to extract. The identification of the torsion distribution is of great importance as it defines the local conformation. As the inter and intramolecular terms of the scattering are merged in the real-space function, a way to deconvolute them is required.

**Figure 9:**
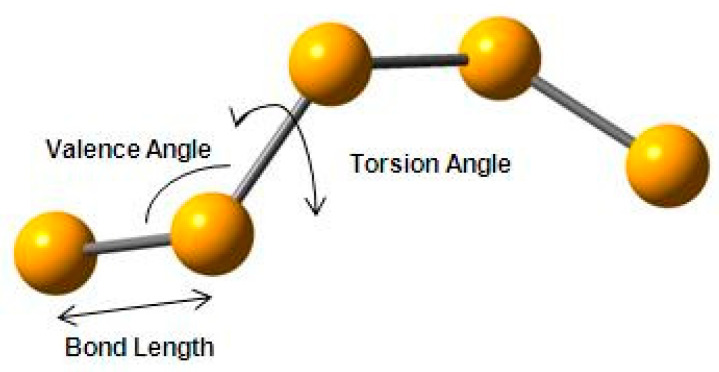
Schematic representation of vitreous selenium indicating the various structural parameters used in the modelling procedure.

This deconvolution can be achieved using the techniques discussed in the previous sections. For this work, we have utilised a method of intramolecular structural analysis that has been developed and used for polymers with very successful results [6,31]. Initially, the model was built by assigning values taken from the literature for bond lengths (2.373 Å), valence angles (103.1°) and torsion angles (±150°) (see schematic in Figure 9). The comparison of the initial model with the experimentally observed structure factor showed us that the general trend and the basic form and shape of the experimental structure factor is being reproduced by the initial model, but refinement of the structural parameters was required in a manner similar to the work in polybutadiene (see Figure 7A) [31].

**Figure 10:**
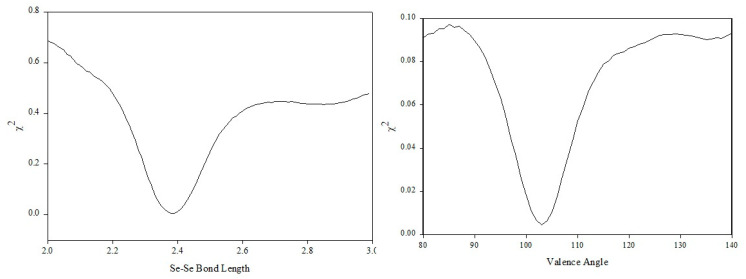
*χ*^2^ test results for the selenium bond length (left) and valence angle (right).

Having built the initial configuration, all parameters are kept constant and the refinement is performed for the bond length. The Se–Se distance is assigned by a Gaussian probability distribution at a particular mean and standard deviation. The position of the distribution is searched initially as it is known that the impact on the scattering curve will be more visible. The model is built and the bond length is assigned via a normal distribution of different lengths at each iteration. Every time the length changes, the scattering pattern is calculated and compared via the statistical test with the experimental one. The result of the search in the case of the bond length can be seen in Figure 10, and a clear minimum exists at a distance of 2.38 Å. Furthermore, the scattering calculated for a bond length of 2.38 Å yields a very good comparison between calculated and observed scattering in high-Q vectors. Consequently, establishing that the oscillating sine wave in the structure factor arises from selenium atoms joins together by covalent bonding. A similar search for the position of the valence angle distribution can be seen in Figure 10. The value of the mean of the distribution of the valence angle was found to be at 105°. The minimum of the search curve was located by identifying the position where the first derivative is equal to zero. A smooth spline curve was fitted to the search result in order to minimise the level of noise, and its first derivative was calculated. Due to the complex nature of the torsion angle distribution, a series of different models were tested and the search was performed by averaging over 100 models at each iteration to ensure better statistics. In the case of the torsion angles, the parameters used in the search were the mean and standard deviation of each state and the relative fraction of the individual states with respect to each other.

#### 7.2.1. First-Order Probabilities

The simplest possible model for the torsion distribution is that where no correlation is assumed between each individual torsion state and its close environment. This yields a picture in which the torsion states are assigned randomly (following specific rules taken from pre-defined distributions) in an unconditional manner having only first-order probabilities and disregarding the previous and following torsion states. That way, two Gaussian distributions were assigned of equal weighting and the position of the torsion distribution was searched in a manner similar to the bond length and valence angle. The distributions were initially assigned at 0° or trans defining the symmetry of the rotation. The search was performed in steps of one degree with one distribution taking values from 0° to −180° and the other from 0° to 180°. Following this procedure, the torsional angle has been detected at a position of ±145°, indicating the existence of a conformation similar to that found in the trigonal structure [59]. The relative fraction of the two distributions was also searched, and we have found out that the probability of a torsion angle followed by one of the same sign (+ + or − −) is almost 100%. This indicates that although the helix is highly distorted due to the valence and torsion angle fluctuations, the energy penalty of the torsion angle to change in sides of rotation is very high.

#### 7.2.2. Conditional Probabilities

In order to include conditional (second-order) probabilities and make the torsion assignment conditional on its neighbours, we have set-up a matrix formulation similar to that used in the RIS model for polymers [1]. That way, the conditional probabilities of the possible helical configurations will be represented as

(12) $$\left(\begin{array}{cc} \alpha & 1-\alpha \\ 1-\alpha & \alpha \end{array}\right)$$

where *α* is the probability of a torsion angle to be followed by one of a same sign. Using the probability α as a parameter in the refinement technique yields a solution of ~1, indicating that long + + + + or − − − − sequences are preferable. This approach of confining the torsional rotations through a Markov Chain representation has been used in the past for a number of polymers like polyethylene [26], PTFE [27] and polybutadiene [6,31].

#### 7.2.3. Predicted Models

**Figure 11:**
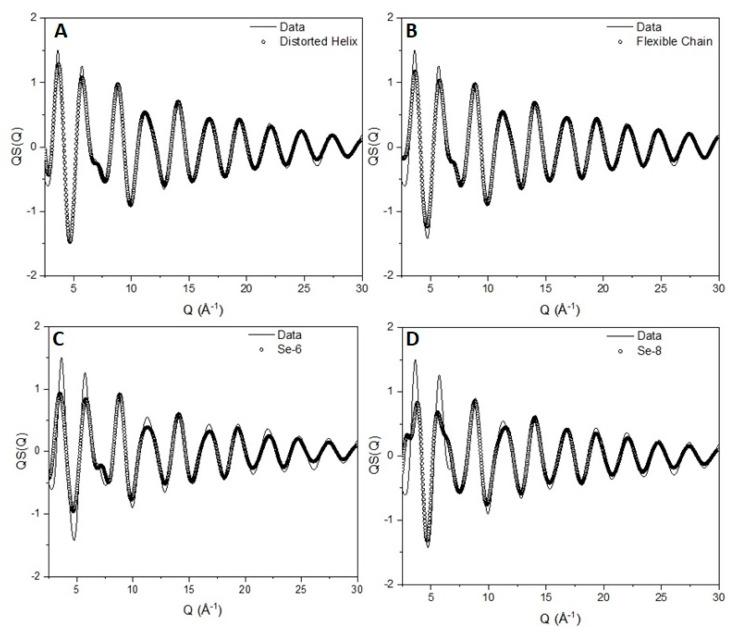
Comparison between the experimentally obtained structure factor (solid line) for vitreous selenium and the scattering pattern obtained from a model of (**A**) a distorted helix, (**B**) a flexible chain, (**C**) six-member rings and (**D**) eight-member rings (open circles).

There have been reports [56] of flat torsional distributions that allow selenium to adopt a highly flexible chain configuration similar to the freely rotating chain model for polymers in solution. We have created a flat torsion angle distribution, keeping bond lengths and angles at their optimum values. The comparison between the experimental data, the ‘random coil’ and the distorted helix can be seen in Figure 11A,B. Although the differences between the two configurations are difficult to observe with the naked eye, the statistical test results show a completely different picture. The χ^2^ test for the helix is 0.0044 ± 0.0001 compared with 0.0065 ± 0.0001 of the ‘random coil’ being at a value 50% larger. Comparison of the structural parameters extracted with the technique presented here with values reported in the literature can be seen in Table S2 in the Supportive Information.

The existence of defects can be handled by inclusion of planar zig-zag trans conformations on the chain. The inclusion of trans states was performed in the same manner as every torsion angle. Initially, trans sequences were included in an unconditional manner in a 3-fold torsion distribution of ±145 and trans (0°), allowing a large probability of − + and + − sequences. The fit was not satisfactory with a *χ*^2^ test of the order of 0.008 significantly larger than the value obtained for the helical chain. Inclusion of conditionality in the probability matrix yields a picture of almost equally probable –trans+ and +trans– sequences with a significantly lower statistical test value of 0.0025 ± 0.0001.

#### 7.2.4. Chain Length

**Figure 12:**
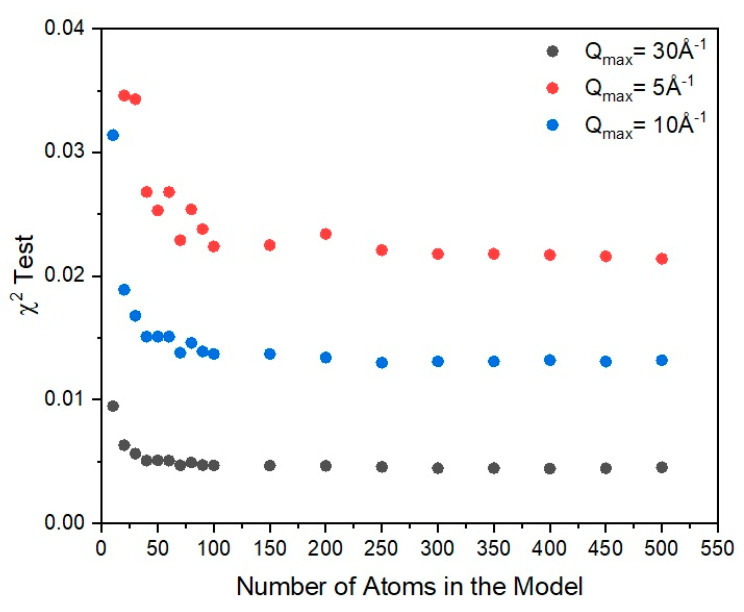
Comparison between the different *χ*^2^ test results for a model of selenium based on the disordered helix as a function of chain length (indicated by the number of atoms in the model) and the different areas of the experimentally observed structure factor. Q_max_ = 30 Å^−1^ indicates the use of the entire scattering curve, Q_max_ = 5 Å^−1^ indicates the use of the first peak of the scattering curve and Q_max_ = 10 Å^−1^ indicates the use of the first two peaks of the scattering curve. In all cases, we can see that the statistical test becomes invariant for a model of at least 100 selenium atoms.

The majority of atoms in amorphous selenium have two-fold coordination, as seen from the radial distribution function analysis. In order to make an approximate estimate on the chain length, we used the scattering pattern to try to establish the level of sensitivity the number of atoms in the model has on the statistical test. Obviously, such an approach provides a rough estimation on the level of information the diffraction pattern carries and its sensitivity on the model size. For this, we used the ″best-fit″ model of the distorted helix. We calculate the scattering of the model and we performed the χ^2^ test in an iterative manner, changing the number of atoms in the model each time. We concluded that based on the scattering data, the diffraction pattern stays almost unaffected by the number of atoms in the chain above *N* > 100. This can be seen in Figure 12 where results of the comparison between similar models of different chain lengths are plotted against the experimentally observed scattering curve. Based on this information, we can say that the model is not sensitive enough to distinguish between models of very large chains. It indicates though that the chains have to be at least 100 atoms long to represent in an adequate way the scattering.

#### 7.2.5. Rings

The presence of six- and eight-member rings has been debated in the past [60], although it has been argued [61] based on optical spectroscopy studies that rings do not appear to any great extent. To investigate the existence of rings, we constructed a model of 1000 six- and eight-member rings, taking the values for their structural characteristics from their relative crystal structure. The comparison between the experimentally observed scattering pattern and the scattering from the rings can be seen in Figure 11C,D. The comparison is clearly not satisfactory especially when compared with the helical-based model (Figure 11B). Adding up the structure factors according to their weighted average leads to a possible fraction of rings in the sample to be less than 2%.

**Figure 13:**
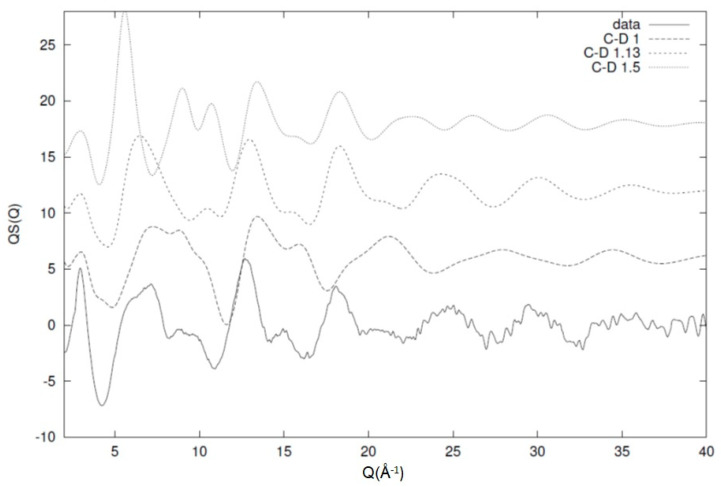
Effect of the carbon–deuterium bond on the overall picture of the diffraction pattern. The best-fit distance of 1.13 Å (short-dashed line) shows a significant resemblance to the experimental data (solid line) when compared with two artificial C-D values of 1 Å (dotted line) and 1.5 Å (short dashed line). All the curves have been shifted for clarity. The image has been reproduced with permission from Reference [6] © American Chemical Society.

From this work, we can see that the model is fairly sensitive and has the ability to distinguish between small variations in the local conformational characteristics and their expression via the diffraction pattern. As discussed previously, similar behaviour has been observed for a number of different polymeric systems [6,26,27,31]. The main reason for this ability is the chain conformation and connectivity that restricts the potential number of configurations that are available to the system. This restriction and deviations from it manifest itself with the existence of unaccounted peaks on the calculated structure factor that are not represented by the experimentally observed one. This has, as a consequence, a nonsatisfactory comparison between the model and the data that leads to a significant increase in the value of the statistical test. An example of this effect can be seen in Figure 13, where the experimentally observed structure factor for a system of deuterated 1,4-polybutadiene is compared with an equivalent model that has different values for the C-D bond length. For 1,4-polybutadiene, the C-D bond length has been estimated from the model at 1.13 Å and, as we can see from Figure 13, the comparison with the experimental data especially in the high-Q region is fairly good. For unrealistic values of the bond length (1 and 1.5 Å in this case), we can see that the comparison between the experimental data and the model prediction is fairly poor [6]. This effect is very pronounced in the case of bond lengths (especially) and valence angles (to a slightly lesser degree) due to the large number of these configurations in the system. Therefore, we can use these structural parameters as a way of excluding unrealistic configurations while keeping the total number of conformations manageable for the model [2].

## 8. Time-Resolved Crystallisation

**Figure 14:**
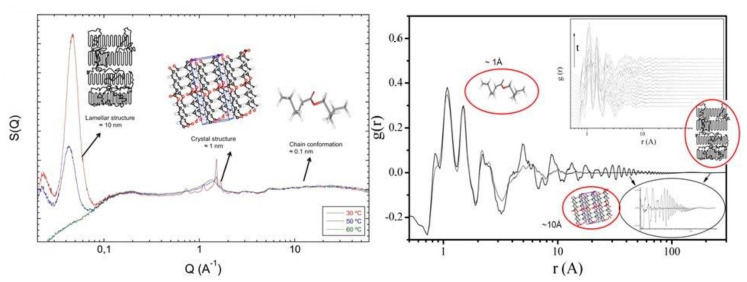
Experimentally observed structure factor (left) and radial distribution function (right) as obtained from a simultaneous small and wide angle neutron scattering experiment in NIMROD. The different length scales associated with the different regions of the functions is highlighted. The inset in the right figure indicates the time evolution of the radial distribution function with temperature.

With modern equipment and increases in time resolution available for neutron diffraction, it is possible to extend the ideas mentioned previously in exploring possibilities of using broad Q diffraction methods in the study of phase transitions such as crystallisation. As the main interest is understanding the molecular-level changes which are involved in this phase transformation, it is clear that unless we have a high-quality model of the melt phase, attempting to identify small deviations from it, in the very early stages, is most likely to lead to no satisfactory conclusion. In Figure 14, we can see the structure factors and radial distribution functions obtained from a system of partially deuterated poly(ε-caprolactone) in the melt and at different processes of crystallization [42,44,45]. The different length scales associated with different parts of the experimentally observed structure factor can be clearly seen and the different features observed in the high-r region of the radial distribution function gives an indication of the power and convenience in utilising the reciprocal space function instead of the real space one. Further analysis of such extended data sets can be made by utilising modified versions of the techniques discussed previously in a sequential manner, starting from the chain conformation and moving towards the coarser length scales. Crystals can be introduced by arranging segments in specified ways while keeping them consistent with the chain conformation, and the overall effect on the scattering pattern can then be studied in an iterative manner [41,42,43,44,45].

## 9. Conclusions

In this work, we have presented an overview of the possibilities for structural analysis offered by intimately coupling high-quality broad *Q* neutron scattering data with atomistic models. Taking advantage of the possibility to separate the structure factor into inter and intrachain contributions allows for the use of reciprocal-space functions instead of the traditional analysis in real space via the radial distribution function. This possibility makes the analysis of the diffraction data unambiguous and eliminates any potential issues arising by terminations and resolution of the Fourier integral.

Atomistic models based on internal parameters that are associated with the polymer structure can be built with a high level of confidence, and the calculation of the relevant scattering function is trivial. Direct comparison of the calculated with the experimentally observed scattering using a statistical test allows a large number of parameters and models to be tested in an iterative way within a very short period of time. Studies on amorphous polyethylene, poly(tetrafluoroethylene), polystyrene, polypropylene and polybutadiene copolymers indicate the robustness of the methodology and offer unique insights on the spatial arrangements of the polymer chains in the bulk. Except the traditional connectivity parameters, these methods allow for extrapolation and identification of large-scale statistical properties like the orientation correlations, the characteristic ratio and the end-to-end length, thus intimately linking the diffraction pattern with unique structural features and correlations as a function of the local conformation and the segmental packing.

Decomposition of the scattering function into partial terms allows for the study of particularly labelled parts of the backbone and bulky side groups, and the way their correlations affect the overall observed diffraction. Experiments in selectively labelled glassy polystyrene give particular insight in the different packing arrangements of the chains and the role played by the phenyl groups reinforcing previously reported ideas based on X-ray diffraction on limited *Q* range. Similar ideas can be used in the study of the extent of mixing in the local level as this is seen through partial structure factors for isostructural blends. Initial studies on selectively labelled polybutadiene blends indicate the role played by the local chain conformation in the extent of mixing. These results are in agreement with previously reported suggestions on the role of the connectivity and local topology on the level of mixing. Using this approach, we can map in a qualitative and quantitative way the different correlations available in the system, and couple them together with the level of local order and see their effect on the overall miscibility, something not possible within the framework of the traditional theory where all these factors are condensed to a single interaction parameter.

## Supplementary Materials

The following are available online at https://www.mdpi.com/2073-4360/12/12/2917/s1, Supplementary information contains: Table S1. Characterisation information for the 1,2 and 1,4 polybutadiene, Table S2 comparison of results of the analysis on vitreous Selenium with previously reported data, preparation methods for the polyethylene materials discussed in the text and the polybutadiene blends.
