# Supplementary material for: The Use of Scattering Data in the Study of the Molecular Organisation of Polymers in the Non-Crystalline State
Source: Polymers (Basel). 2020 Dec 5;12(12):2917. doi: 10.3390/polym12122917 (PMC7762082; doi:10.3390/polym12122917)
Supplement: Supplementary file 1 [file polymers-12-02917-s001.pdf]

polymers

MDPI

*Supplementary*

# The Use of Scattering Data in the Study of the Molecular Organisation of Polymers in the Non-Crystalline state

**Thomas Gkourmpis [1]* and Geoffrey R. Mitchell [2]**

[1] Innovation & Technology, Borealis AB, Stenungsund, SE-444 86, Sweden; Thomas.gkourmpis@borealisgroup.com
[2] Centre of Rapid and Sustainable Product Development, Institute Polytechnic Leiria, Marinha Grande, Portugal; Geoffrey.mitchell@ipleiria.pt
* Correspondence: Thomas.gkourmpis@borealisgroup.com; Tel.: +46 303 205 576

Received: date; Accepted: date; Published: date

## Supplementary Information

Characterisation information for the different materials used in this study can be seen in Table 1

| Material | $M_w$ | $M_n$ | $M_w/M_n$ | 1,2 content (%) | 1,4 Content (%) | $T_g$ (°C) |
|---|---|---|---|---|---|---|
| **d-1,4 PBD** | 75000 | 79500 | 1.06 | 7 | 93 | -103 |
| **d-1,2 PBD** | 72000 | 77700 | 1.08 | 93 | 7 | -13.5 |
| **h-1,4 PBD** | 84000 | 87360 | 1.04 | 7 | 93 | -100 |
| **h-1,2 PBD** | 75300 | 81300 | 1.08 | 93 | 7 | -14 |

**Table S1.** Characterisation information for the 1,4 and 1,2-polybutadiene (PBD) systems used in this study. The indices d and h stand for deuterated and protonated systems respectively.

## Polybutadiene Blends Sample Preparation and Experimental Procedure

Fully deuterated (>99%) 1,4-polybutadiene and 1,2-polybutadiene was obtained by Polymersource Inc. in Canada. Protonated 1,4-polybutadiene and 1,2-polybutadiene was also obtained by Polymersource Inc. in Canada. Material characterization can be seen in Table 1.

Each sample was dissolved in cyclohexane and casted in a container made of aluminum of 1mm thickness and 36mm diameter. All samples were dried in atmospheric pressure followed by vacuum for 48h and the evaporation of the solvent was checked by weighting. As soon as no changes in weight were observed the samples were assumed that had the majority of the solvent extracted.

Neutron scattering experiments were performed in ISIS Pulsed Neutron Source in the UK using SANDALS Diffractometer. Temperature was controlled by a CCR cryogenic unit with temperature fluctuations of the order of ±2°C. All data were collected for a minimum of 500μA to a maximum of 3,500μA integrated proton current to ensure reasonable signal to noise ratio. Data were collected in all detector banks and normalized to the incident neutron beam and calibrated by a vanadium standard.

## Polyethylene Sample Preparation and Experimental Procedure

Fully deuterated (>99%) polyethylene ($M_n$=109,000, $M_w/M_n$=1.02) was obtained by Polymersource Inc. in Canada. Sample was dissolved in Toluene and cast into a thin film of 40mm diameter and 1mm thickness. Solvent evaporation was checked by constant weighting at room temperature (~24 hours) and in vacuum (48 hours). After constant weight was reached it was assumed all solvent molecules have been extracted.

Neutron scattering experiments were conducted in ISIS Pulsed Neutron Source in the UK using SANDALS Diffractometer. Data were collected to 1,000μA integrated proton current, in all detector banks and were normalized to the incident neutron beam using a vanadium standard.

| **Method** | ***l*** **(Å)** | **φ (deg)** | **N** | ***θ*** **(deg)** | **Ref** |
|---|---|---|---|---|---|
| This work | 2.38±0.024 | 103±3.5 | | 85±5 | |
| RDF Analysis* | 2.346±0.013 | 104±5 | 1.974±0.205 | | [1], [2] |
| MD Simulation | 2.35 | 106 | | 90±40 | [3] |
| MD Simulation | 2.3 | 103 | 2.1 | | [4] |
| MD Simulation | 2.37 | 102.8 | | 92.9 | [5] |
| MD Simulation | 2.37 | 102 | | 100 | [6] |
| MD Simulation | 2.38-2.36 | 104-107±10-19 | 2-1.88 | 90 | [7] [8] |
| MD Simulation | 2.37 | 103 | 1±0.4 2±0.4 3±0.4 | | [9] |
| MD Simulation | | 103 | 2 (82%-83%) 3 (9-8.5%) | 90 | [10] |
| MD Simulation | 2.36 | 106 | 2 (71%) 3 (18%) | Uniform | [11] |
| Disordered Chain | 2.3-2.4 | 103-106 | | 102 | [12, 13] |

**Table S2**. Comparison between results obtained from this work and previously reported theoretical predictions for the structure of Vitreous Selenium. $l$ corresponds to the bond length, $\varphi$ to the valence angle, $\vartheta$ to the torsion angle and N to the coordination number.

## References


1. Wright, A., *Experimental Techniques of Glass Science, ch. 8.* Westerville, Ohio: American Ceramic Society, 1993: p. 205.
2. A. C. Wright, C.A.G., R. A. Hulme, S. W. Martin, R. N. Sinclair. *Proc. 14th Conf. on Glass and Ceramics.* 2005.
3. Nakamura, K. and A. Ikawa, *Medium-range order in amorphous selenium: Molecular dynamics simulations.* Physical Review B, 2003. **67**(10): p. 104203.
4. Caprion, D. and H.R. Schober, *Computer simulation of liquid and amorphous selenium.* Journal of Non-Crystalline Solids, 2003. **326-327**: p. 369-373.
5. Almarza, N.G., E. Enciso, and F.J. Bermejo, *Structure and dynamics of selenium chain melts: A molecular dynamics study.* The Journal of Chemical Physics, 1993. **99**(9): p. 6876-6889.
6. Hohl, D. and R.O. Jones, *First-principles molecular-dynamics simulation of liquid and amorphous selenium.* Physical Review B, 1991. **43**(5): p. 3856-3870.

7. Kirchhoff, F., G. Kresse, and M.J. Gillan, *Structure and dynamics of liquid selenium.* Physical Review B, 1998. **57**(17): p. 10482-10495.
8. Kresse, G., F. Kirchhoff, and M.J. Gillan, *Defects in liquid selenium.* Physical Review B, 1999. **59**(5): p. 3501-3513.
9. Molina, D., E. Lomba, and G. Kahl, *Tight-binding model of selenium disordered phases.* Physical Review B, 1999. **60**(9): p. 6372-6382.
10. Shimizu, F., et al., *Chain structure of liquid and amorphous selenium: tight-binding molecular-dynamics simulation.* Journal of Non-Crystalline Solids, 1999. **250-252**: p. 433-436.
11. Bichara, C., A. Pellegatti, and J.P. Gaspard, *Chain structure of liquid selenium investigated by a tight-binding Monte Carlo simulation.* Physical Review B, 1994. **49**(10): p. 6581-6586.
12. Misawa, M. and K. Suzuki, *Structure of Chain Molecule in Liquid Selenium by Time-of-Flight Pulsed Neutron Diffraction.* Transactions of the Japan Institute of Metals, 1977. **18**(5): p. 427-434.
13. Misawa, M. and K. Suzuki, *Ring-Chain Transition in Liquid Selenium by a Disordered Chain Model.* Journal of the Physical Society of Japan, 1978. **44**(5): p. 1612-1618.

© 2020 by the authors. Submitted for possible open access publication under the terms and conditions of the Creative Commons Attribution (CC BY) license (http://creativecommons.org/licenses/by/4.0/).
